# Cost Savings of Using Updated Thai Red Cross Intradermal Regimen in a High-Throughput Anti-Rabies Clinic in New Delhi, India

**DOI:** 10.3390/tropicalmed4010050

**Published:** 2019-03-22

**Authors:** Bijit Kumar Kundu, Girish Gulab Meshram, Shrinath Bhargava, Omprakash Meena

**Affiliations:** 1 Department of Medicine, Postgraduate Institute of Medical Education and Research and Dr. Ram Manohar Lohia Hospital, New Delhi 110001, India; bijit1973@gmail.com; 2 Department of Pharmacology, Postgraduate Institute of Medical Education and Research and Dr. Ram Manohar Lohia Hospital, New Delhi 110001, India; 3 Department of Dermatology, Postgraduate Institute of Medical Education and Research and Dr. Ram Manohar Lohia Hospital, New Delhi 110001, India; sbderma60@hotmail.com; 4 Department of Orthopedics, Postgraduate Institute of Medical Education and Research and Dr. Ram Manohar Lohia Hospital, New Delhi 110001, India; dropmeena1978@gmail.com

**Keywords:** rabies, post-exposure prophylaxis, Essen regimen, updated Thai Red Cross regimen, cost savings

## Abstract

Replacement of the Essen intramuscular (EIM) by the updated Thai Red Cross intradermal (UTRCID) regimen for rabies post-exposure prophylaxis (PEP), in high-throughput hospitals of India, has been advocated since 2006 thanks to its cost-effectiveness. However, several anti-rabies clinics in India and other parts of the world have not initiated this switchover of regimens because of the paucity of financial literature, generated in realistic settings, regarding the same. We calculated the procurement costs of various items required for providing rabies vaccinations via the EIM regimen and UTRCID regimen, on an annual basis, a year before and after the switchover. From a healthcare provider’s perspective, the cost of vaccination per patient was calculated to be 5.60 USD for the EIM regimen and 2.40 USD for the UTRCID regimen. The switchover to the UTRCID regimen from the EIM regimen reduced the financial burden of the rabies vaccination by almost 60%. Procurement of vaccine vials contributed to the majority of the cost (>94%) in both of the regimens. Procurement of syringes with fixed needles contributed negligibly (<6%) to the financial burden in both the regimens. A policy to progressively switch over to the UTRCID regimen from the EIM in all high-throughput anti-rabies centers of India would dramatically reduce the economic burden of running a successful anti-rabies program.

## 1. Introduction

Rabies is one of the oldest and most dreaded zoonotic disease known to mankind. The rabies virus enters the human body via bites of rabid animals and causes an acute, progressive, and fatal encephalomyelitis [1,2]. The global cost of rabies has been estimated to be 8.6 billion USD and causes the loss of over 3.7 million disability-adjusted life years [3]. India accounts for 35% of all deaths due to rabies. In India, every two seconds, a person is bitten by a rabid animal, and every 30 min, a person dies of rabies [4]. Because rabies is not a notifiable disease in India and there is no organized surveillance system of human cases, the abovementioned burden is a gross underestimate [4,5]. 

In India, dog bites account for majority of the cases of human rabies. Children and people of lower socio-economic status are the most vulnerable population because of their higher exposure to stray dogs [5]. India has about 60 million dogs, the vaccination status of which is unknown [6]. Although rabies is 100% fatal once symptoms develop, it can be prevented through prompt post-exposure prophylaxis (PEP). PEP consists of effective washing of the wounds that occurred during exposure, administration of rabies immunoglobulin (if indicated), and administration of an effective rabies vaccine [7]. 

For decades, only the nerve tissue rabies vaccine was available in India for rabies PEP, which caused neuroparalysis as an adverse effect [8]. In 2004, the nerve tissue vaccine was banned in India, which provided the gateway to the safer and more efficacious cell culture rabies vaccine [9]. However, as the cell culture rabies vaccine was more expensive then, the period between 2004 and 2006 was marked by shortage or absence of vaccines in most anti-rabies clinics of New Delhi, India. In that period, the rabies vaccine was administered according to the Essen intramuscular (EIM) regimen in both low- and high-throughput anti-rabies centers, catering to patients of animal bites [8,9]. In 2006, as evidence regarding the efficacy of various intradermal regimens began to mount through rigorous clinical trials, the updated Thai Red Cross intradermal (UTRCID) regimen was approved for rabies PEP in India by the Drug Controller General of India (DCGI), which is the drug and vaccine regulatory authority of India [5]. This approval occurred 14 years after the World Health Organization (WHO) had recommended the UTRCID regimen [5,10]. 

On the basis of new clinical evidence and public health needs, WHO recently recommended the dose-sparing abridged one-week intradermal regimen for rabies PEP [11,12]. A study utilizing epidemiological and economic models projected that a switchover to the dose-sparing abridged one-week intradermal regimen along with Gavi (The Vaccine Alliance) investment for improving PEP accessibility and free provision would cost-effectively reduce the disease burden without much change in the vaccine requirement [13]. However, the dose-sparing abridged one-week intradermal regimen is yet to be approved by the DCGI for routine use in India. As of now, the EIM and UTRCID regimens are the only two approved regimens for providing rabies vaccination in India [14].

As the volume of vaccine required in the UTRCID regimen is much lower than that of the EIM regimen, several reports have suggested that the UTRCID regimen is 60–80% more economical than the EIM regimen [5,15]. However, these estimations are not backed by hospital-generated financial data. Moreover, most of the cost-effectiveness studies regarding various rabies vaccination regimens have been conducted in hypothetical settings from a societal point of view, making them of limited use to policy makers [16,17]. 

Despite almost a decade following the introduction of the UTRCID regimen in India, several anti-rabies centers in India have not initiated the switchover to the UTRCID regimen from the EIM regimen [8]. Cost savings is an impactful driver for policy makers for creating policies to galvanize this switchover of regimens. Hence, we conducted this retrospective observational study to determine the quantum of benefit accrued by our hospital, following the replacement of the EIM regimen by the UTRCID regimen, from a healthcare provider’s perspective.

## 2. Materials and Methods

### 2.1. Place of Study

This retrospective observational study was conducted in the anti-rabies clinic of our hospital, after approval of the institutional ethics committee. Our hospital is a 1420-bedded tertiary healthcare center governed by the Central Government of India via the Ministry of Health and Family Welfare. Approximately 2 million patients visit our outpatient department annually, of which around 30,000 cases are of animal bites. The anti-rabies clinic of our hospital functions from 09:00 to 16:00 on all weekdays and from 09:00 to 13:00 on Saturdays. 

### 2.2. Determination of Observation Period 

The EIM regimen was the only regimen followed for the administration of the rabies vaccine in the anti-rabies clinic of our hospital until 2010. The switchover from the EIM regimen to the UTRCID regimen was initiated in our anti-rabies clinic from April 2011 and was completed by July 2011. Following the switchover of regimens, all patients visiting our anti-rabies clinic received rabies vaccination only via the UTRCID regimen. In other words, patients vaccinated in the year 2010 were exclusively vaccinated via the EIM regimen and patients vaccinated in the year 2012 were exclusively vaccinated via the UTRCID regimen. Hence, in order to avoid an overlap of regimens and to minimize errors due to population variation, the years 2010 and 2012 were selected to calculate the economic benefit accrued by our hospital, following the switchover of regimens. 

### 2.3. Inclusion and Exclusion Criteria

Case records of all patients visiting the anti-rabies clinic of our hospital with history of animal bite in the years 2010 and 2012 were included in the study, irrespective of age or gender. Anti-rabies serum is expensive and is often procured in smaller quantities by most low-throughput anti-rabies clinics located in surrounding areas of our hospital. When stockouts of anti-rabies serum occur in such low-throughput anti-rabies clinics, vaccinated patients are often referred to high-throughput anti-rabies clinics such as ours for exclusive administration of anti-rabies serum. Such patients who had visited our anti-rabies clinic for exclusive administration of anti-rabies serum injections without the need for vaccination were excluded from analysis.

### 2.4. Determination of Seasonal Variability

In order to determine whether a seasonal variation existed in our included study population, we represented the patient load data biannually for both the years, wherein the months from January to June were considered as the spring and summer months and the months from June to December were considered as the autumn and winter months. 

### 2.5. Rabies Vaccines Provided

Vaccine vials for both the regimens were of 1 mL volume and were stored at +2 °C to +8 °C. Intramuscular rabies vaccine (brand name: ChengDa) was procured from Liaoning Cheng Da Biologicals Co. Ltd., Shenyang, China. Intradermal rabies vaccine (brand name: Abhayrab) was procured from Indian Biologicals Institute, Hyderabad, India. The potency of each of the vaccines was ≥2.5 IU per vial, as per the set WHO standards. Numerous studies have shown that WHO-recommended vaccines (≥2.5 IU per intramuscular dose), when administered by the intradermal route for PEP, have efficacy equivalent to or higher than that of the same vaccine administered by the intramuscular route [11,18]. Hence, we considered both the vaccines to be of similar efficacy in our study. 

PEP was administered by the nursing staff, who were well-trained to inject vaccines via both the intramuscular and intradermal routes. The sensitization of the nursing staff regarding both the regimens was done by the medical officers posted in the clinic from time to time. The following standard operating procedures were followed by the nursing staff, while administrating the rabies vaccine via the EIM and UTRCID regimens. 

#### 2.5.1. EIM Regimen

The area of the injection site was cleaned with cotton dipped in 70% alcohol. A 1 mL syringe with a fixed needle was loaded with 1 mL of the purified vero cell rabies vaccine. As per the EIM regimen (1-1-1-1-1), 1 mL of the vaccine was injected into the deltoid muscle on days 0, 3, 7, 14, and 28. 

#### 2.5.2. UTRCID Regimen 

Similar to the EIM regimen, the injection sites were cleaned with cotton dipped in 70% alcohol. Following which, 0.2 mL of the reconstituted vaccine was drawn into a 1 mL syringe with a fixed needle. As per the UTRCID regimen (2-2-2-0-2), 0.1 mL of the reconstituted vaccine was injected intradermally at the deltoid area of each arm on days 0, 3, 7, and 28. Following each intradermal injection, a raised and palpable bleb of around 5 mm in diameter with peau d’ orange appearance was considered indicative of correct technique followed. After the injection, the injection site was left open without any dressing. Patients were instructed to not rub or apply any antiseptic at the injection site. The remainder of the vaccine in the vial was used for the next patient. Content remaining in the vial after 6 h was discarded.

### 2.6. Cost Estimation

The consumption of rabies vaccine vials and syringes with fixed needles by the anti-rabies clinic of our hospital was considered for calculating the cost of vaccination for each of the regimens. Costs due to 70% alcohol (antiseptic) and cotton procurement were excluded from analysis as they were negligible. Financial liabilities due to the services of the medical/paramedical staff were excluded from analysis, as their salaries remained the same in the observed time period. We assumed that the cost savings that would arise from the switchover would be the result of the difference in the cost of vaccination of both the regimens. Hence, we did not calculate the costs pertaining to other aspects of providing PEP such as washing the bite area with soap, application of antiseptic, and injection of human anti-rabies serum. Furthermore, management guidelines were the same regarding these aspects, irrespective of the regimen. 

Data regarding the annual consumption pattern of various items by the anti-rabies clinic of our hospital, in the specified time period, were obtained from the inventory management section of our hospital. Data regarding the number of patients visiting the anti-rabies clinic of our hospital were obtained from the medical records section of our hospital, which were vetted by the staff of the anti-rabies clinic. The rates of all the items with taxes were obtained from the procurement section of our hospital. Procurement of all items in our hospital was done via the tendering process, because of which the approved rates of all items, considered for cost calculation, varied on an annual basis in the observed time span. Hence, in order to bring uniformity in the data, we adjusted the prices of all the items, as per their current approved rates in our hospital for the financial year 2018–2019. Only the adjusted costs were considered for final analysis and were discussed. The cost was calculated in Indian National Rupees and converted to USD, as per historical conversion rates of 2010, 2012, and 2018. As ours is a government-run hospital, all patients visiting our center were not charged for any of the items or services required for rabies PEP. 

### 2.7. Formulae Used for the Calculation of Various Variables

The cost of vaccination ($C_{R}$) for the EIM regimen and UTRCID regimen was calculated separately as follows:

(1) $$C_{R}=\left(n_{v}\;\times \;C_{v}\right)\;+\left(n_{s}\;\times \;C_{s}\right),$$

where $n_{v}$ and $n_{s}$ are the number of vaccine vials and syringes with fixed needles consumed, respectively. $C_{v}$ and $C_{s}$ are the costs per unit of vaccine vials and syringes with fixed needles, respectively (as per hospital-approved 2018–2019 rates). 

Percentage cost savings following the switchover of regimens ($C_{D}$) was calculated as follows:

(2) $$C_{D}=\;\left(\frac{C_{R\left(EIM\right)}}{n_{R\left(EIM\right)}}-\frac{C_{R\;\left(UTRCID\right)}}{n_{\;R\left(UTCRID\right)}}\right)\;\times \;100,$$

where $C_{R\left(EIM\right)}$ and $C_{R\left(UTRCID\right)}$ are the costs of vaccination for the EIM regimen and UTCRID regimen, respectively. $n_{R\left(EIM\right)}$ and $n_{R\left(UTCRID\right)}$ are the number of patients vaccinated via the EIM regimen and UTRCID regimen, respectively. 

Difference in the consumption rate of vaccine vials per patient ($P_{v}$), following the switchover of regimens, was calculated as follows:

(3) $$P_{v}=\left(\frac{n_{v\;\left(EIM\right)}}{n_{R\;\left(EIM\right)}}-\frac{n_{v\;\left(UTRCID\right)}}{n_{R\;\left(UTRCID\right)}}\right)\;\times \;100,$$

where $n_{v\;\left(EIM\right)}$ and $n_{v\;\left(UTRCID\right)}$ are the number of vaccine vials consumed by patients vaccinated via the EIM regimen and UTRCID regimen, respectively.

Difference in the consumption rate of syringes with fixed needles per patient ($P_{s}$), following the switchover, was calculated as follows:

(4) $$P_{s}=\left(\frac{n_{s\;\left(UTRCID\right)}}{n_{R\;\left(UTRCID\right)}}-\frac{n_{s\;\left(EIM\right)}}{n_{R\left(EIM\right)}}\right)\;\times \;100,$$

where $n_{s\;\left(EIM\right)}$ and $n_{s\;\left(UTRCID\right)}$ are the number of syringes with fixed needles consumed by patients vaccinated via the EIM regimen and UTRCID regimen, respectively. 

### 2.8. Statistical Analysis 

We conducted a descriptive statistical analysis of the data. As all the variables in our study were quantitative, we expressed the data as percentages, as and when required. 

## 3. Results

A total of 22,049 cases of animal bite visited the anti-rabies clinic of our hospital in the year 2010, of which 12,619 cases were vaccinated. The remaining cases had visited the anti-rabies clinic for exclusive administration of anti-rabies serum without vaccination. In the year 2012, 30,630 patients of animal bite visited the anti-rabies clinic of our hospital, of which 16,904 cases were vaccinated and fitted the inclusion criteria. Hence, 12,619 and 16,904 patients were considered to have been provided rabies vaccination via the EIM regimen and UTRCID regimen, respectively, in the observation period of the study. 

**Figure 1:**
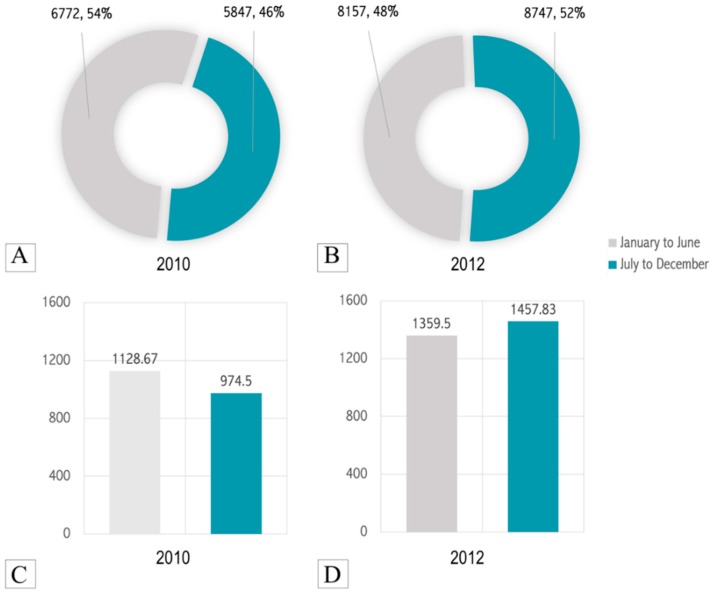
Biannual/seasonal distribution of vaccinated patients (n, %) in 2010 (**A**) and 2012 (**B**). Average number of patients vaccinated per month (n) in each half of 2010 (**C**) and 2012 (**D**).

Of the 12,619 cases vaccinated in 2010, 6772 cases were vaccinated in the spring and summer months (January to June) and 5847 cases were vaccinated in the autumn and winter months (July to December). Hence, on average, 1128.67 and 974.5 cases were vaccinated on a monthly basis in the first half and second half of 2010, respectively (Figure 1). Similarly, of the 16,904 patients vaccinated in 2012, 8157 cases were vaccinated in the spring and summer months (January to June) and 8747 cases were vaccinated in the autumn and winter months (July to December). Hence, on average, 1359.5 and 1457.83 cases were vaccinated on a monthly basis in the first half and second half of 2012, respectively (Figure 1). 

**Figure 2:**
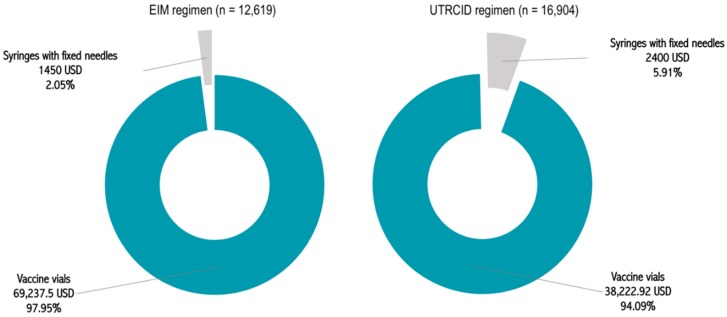
Costs borne by our hospital for providing rabies vaccination for a period of one year via the Essen intramuscular (EIM) regimen and updated Thai Red Cross intradermal (UTRCID) regimen. Costs are expressed in USD. n = number of patients.

**Figure 3:**
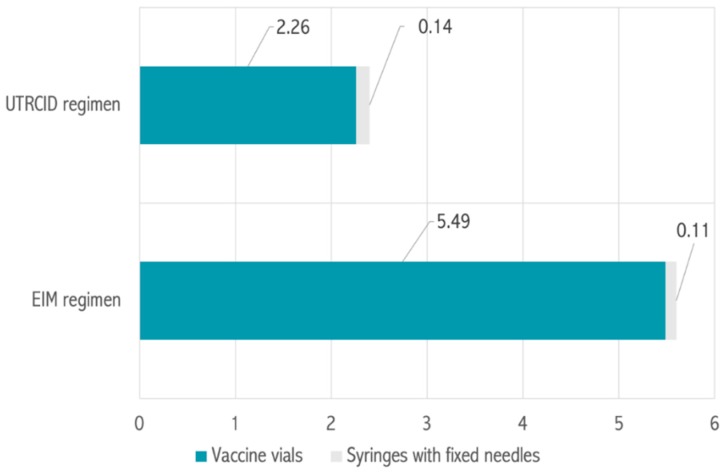
Cost per person for rabies vaccination via the Essen intramuscular (EIM) regimen and updated Thai Red Cross intradermal (UTRCID) regimen. Costs are expressed in USD.

After adjusting the cost of every item, as per their approved rates for the financial year 2018–2019, the total cost borne by our hospital for providing the rabies vaccination was estimated to be 70,687.5 USD for the EIM regimen and 40,622.92 USD for the UTRCID regimen (Figure 2, Table 1). Accordingly, the cost of vaccination per patient was calculated to be 5.60 USD for the EIM regimen and 2.40 USD for the UTRCID regimen, a reduction of 57.14% (Table 1, Figure 3). 

**Figure 4:**
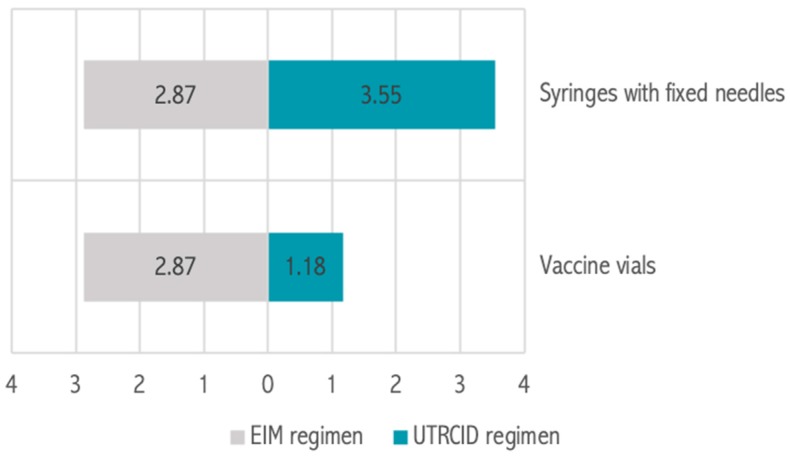
Items consumed per person for rabies vaccination via the Essen intramuscular (EIM) regimen and updated Thai Red Cross intradermal (UTRCID) regimen. Values are expressed as *n*.

The EIM regimen and UTRCID regimen cohorts consumed 36,250 and 20,012 vaccine vials, respectively. The vaccine vial consumption rate per patient was calculated to be 2.87 vials for the EIM regimen and 1.18 vials for the UTRCID regimen, a reduction of 58.89% (Figure 4). Vaccine vials contributed to 97.95% and 94.09% of the total procurement cost of providing rabies vaccination via the EIM regimen and UTRCID regimen, respectively (Figure 2 and Figure 3).

Syringes with fixed needles contributed to 2.05% and 5.91% of the total expenditure of vaccinating patients via the EIM regimen and UTRCID regimen, respectively (Figure 1). The syringe with fixed needle consumption rate was 2.87 for the EIM regimen and 3.55 per patient for the UTRCID regimen, an increase of 19.14% (Figure 4). 

## 4. Discussion

Our study determined that switching over to the UTRCID regimen from the EIM regimen, in a high-throughput anti-rabies clinic such as ours, reduced the cost of vaccination by almost 60%. Our findings are consistent with the hypothetical projections, described in earlier reports [5,15]. Procurement of vaccine vials contributed maximally to the cost of vaccination, in both the regimens. Following the switchover, the vaccine vial consumption rate per patient was drastically reduced, as reported previously [9]. Syringes with fixed needles contributed negligibly to the cost of vaccination, in both the regimens. 

Annually, around 17 million people are exposed to rabies worldwide, more than 15 million people receive PEP, around 60,000 humans die as a result of rabies, over 3.7 million disability adjusted life years are lost, and more than 8.6 billion USD is spent in rabies control programs [4,9,19]. The bulk of the global burden of rabies lies in low- and middle-income countries including India [20,21]. Despite considerable research on rabies in Asia and Africa, there lies a research–policy disconnect leading to a lack of implementation of effective rabies-control policies [16,17,21]. This is evident by the rising incidence of rabies, and numerous centers still continuing with costlier intramuscular regimens [19,22].

Progressively switching over to cost-effective WHO-recommended intradermal PEP regimens, improving PEP availability and accessibility, scaled-up mass canine vaccination, and heightened public awareness of rabies PEP are suggested as central policies for eliminating human deaths from dog-mediated rabies [10,12,13]. Economic gains made by healthcare providers via the reduction of procurement costs is a major driver for policy makers for implementing effective policies [10,13]. 

The EIM and UTRCID are the only two regimens for rabies vaccination approved by the drug regulatory authority of India [14]. The UTRCID regimen reduces the volume of the vaccine required, as compared with the EIM regimen. On the basis of this fact, several reports have projected that the overall cost of providing PEP per patient is reduced by 60–80%, following the switchover of regimens [23,24]. However, these projections of cost reduction have been estimated from a patient’s perspective in hypothetical settings and have not included other expenses such as procurement of syringes/needles and so on. In realistic settings, from the perspective of a healthcare provider, the cost of providing rabies vaccination is reduced by almost 60% by the UTRCID regimen, in a high-throughput anti-rabies center, as estimated in our study. The reduction in the overall cost of providing rabies vaccination could reduce the incidents of vaccine shortage, which are fairly common in economically-deprived nations [4,8]. Also, in vaccine-sufficient clinics, these economic gains could be diverted towards the procurement of anti-rabies serum vials, which also are perennially in limited supply thanks to their exorbitant cost [24]. Switching over to the UTRCID regimen from the EIM regimen would also improve the equity and coverage of vaccination, and reduce both direct and indirect costs to patients. Also, overcrowding of patients at high-throughput anti-rabies clinics would be substantially reduced [12].

Ideally, each naïve case of animal bite should consume 5 ml of the vaccine via the EIM regimen and 0.8 mL of the vaccine via the UTRCID regimen, if all the doses are received. In our study, we observed that each patient receiving vaccination via the EIM regimen received only 2.87 mL of the vaccine, which translates to around 2–3 doses. The vaccine consumption lower than the total dose was observed as follow-up and previously vaccinated patients in other anti-rabies centers were also included in the study population. Also, patients may have discontinued vaccination because of reasons such as the biting animal being healthy after ten days of observation, and factors causing non-compliance such as loss of wages, distance from hospital, and so on [25]. On the contrary, the vaccine consumption rate of 1.18 ml per case via the UTRCID regimen was much higher than the total recommended dose. This observation could be explained by the higher vaccine wastage rate in the UTRCID regimen, as partially used vials were discarded to minimize the risk of bacterial contamination. Also, previous studies have documented that the compliance rate for the UTRCID regimen is much higher than that for the EIM regimen [22,25]. A study conducted at a smaller anti-rabies center in India, delivering rabies vaccination via the UTRCID regimen, reported a vaccine vial consumption rate of 0.7 vials per person, including the wastage [9]. The lower vaccine consumption rate in smaller anti-rabies centers could be the result of the fact that smaller anti-rabies centers are more tightly regulated and do not operate on a daily basis. Despite assuming a higher vaccine wastage rate in the URTCID regimen, the overall economic gains made in the URTCID regimen were much higher than in the EIM regimen, in a high-throughput hospital such as ours.

Assuming complete utilization of the vaccine vials, our hospital inventory section dispensed one and four to five syringes with fixed needles per vaccine vial for the EIM regimen and UTRCID regimen, respectively. The syringe with fixed needle consumption rate was slightly higher in the UTRCID regimen, as compared with the EIM regimen. However, we observed that syringes with fixed needles, contributed to only a minor fraction (<6%) of the overall cost, in both the regimens. However, anti-rabies clinics planning to switchover to the UTRCID regimen and planning to administer each dose, that is, 0.1 mL by a fresh syringe/needle, should consider the cost of procuring syringes and needles beforehand. Preferably, a separate syringe should be used for each intradermal dose of the UTRCID regimen [23]. Although we provided both the intradermal doses per visit via one syringe, we followed strict aseptic techniques to avoid injection site infections. Also, we preferred syringes with fixed needles in order to avoid vaccine wastage. As procurement of the vaccine vials contributed maximally to the cost of vaccination, it should be the primary decisive factor considered before the switchover of regimens. 

A previous cost–analysis study, examining the cost-effectiveness of various rabies vaccination regimens in various hospital settings (from low- to high-throughput anti-rabies clinics), concluded that higher the patient throughput at an anti-rabies clinic, the higher the cost-effectiveness of intradermal vaccination regimens. The same study projected that a switchover from an intramuscular regimen to an intradermal regimen at an anti-rabies clinic with less than 10 new patients per month could reduce the vaccine usage by 25% [26]. Consistent with this projection, a study conducted at a primary healthcare center in India, catering to around six animal bite cases per week, administered the rabies vaccine via the UTRCID regimen with a vaccine vial wastage rate of around 20% [9]. However, some reports have also suggested that smaller and peripheral anti-rabies centers in India, receiving fewer number of patients, that is, one to two patients per week, may not benefit from the switchover of regimens as factors such as costs for training/supervising the staff, and high wastage rates could overshadow the economic gains made as a result of lower procurement costs [16,17,21]. A recent report recommended that any anti-rabies clinic with at least four patients of animal bite per day (naïve and repeat cases) should implement the UTRCID regimen in order to minimize vaccine wastage [10]. Hence, considering the disparity regarding the choice of regimen for rabies vaccination in low-throughput anti-rabies clinics, more comprehensive financial studies stratifying various small/peripheral anti-rabies centers according to their patient load on a daily/weekly basis could clarify the exactness of the economic advantages, if any, after implementation of the UTRCID regimen.

Vaccine wastage is one of the major concerns of implementing the UTRCID regimen in both high- and low-throughput anti-rabies clinics [16,26]. Several measures could be put in place in order to reduce wastage such as if the patient count is low on some days, the remainder of the vial could be used for pre-exposure prophylaxis [10]. Development of appropriate, inexpensive devices and pre-filled syringes to facilitate vaccination could reduce wastage along with reducing the financial burden of training vaccinators [10,26]. Clinical trials in order to reduce the dosage or the number of visits of patients in intradermal regimens are underway [27]. Research into methods of preserving rabies vaccines and preventing contamination could further enable economic use of vaccine vials [26]. 

The newly WHO-recommended dose-sparing abridged one-week intradermal regimen (2-2-2), also called the Institut Pasteur du Cambodge (IPC) regimen, is suggested to be even more cost-effective than the UTRCID regimen [11]. Replacement of the UTRCID regimen by the dose-sparing abridged one-week intradermal regimen would further reduce vaccine usage per patient by 25%, allowing the treatment of 33% more patients with the same quantity of vaccine [7,12]. A recent study utilizing multilayer mathematical models projected that a switchover to the dose-sparing abridged one-week intradermal regimen along with Gavi investment for improving PEP accessibility and availability would cost-effectively avert costs of 635 USD per death and 33 USD per disability-adjusted life years. Also, 17.4 million more patients would be vaccinated with a similar vaccine requirement [13]. However, India and several other countries would have to gain the approval of their respective drug/vaccine regulatory agencies before reaping the benefits of this new WHO-recommended intradermal regimen. Currently, WHO-recommended intradermal regimens have not been approved in several countries including China, Malaysia, Taiwan, and Indonesia [14]. 

Studies in several countries have reported a seasonal variability in animal bite patients, that is, a higher number of cases occur in summer and spring months [28,29]. However, in the two years that we analysed, we could not elucidate a considerable and consistent increase/decrease in the vaccinated patients on a biannual/seasonal basis, that is, the proportions of vaccinated patients were almost similar in both halves of the year. As the inflow of patients to most high-throughput anti-rabies clinics is influenced by several heterogenous factors such as stockouts of vaccines in nearby low-throughput clinics, it often becomes very difficult to isolate and correlate each of these factors causing an increase or decrease in the patient load [29]. Future longitudinal epidemiological studies pooling data from several anti-rabies clinics in and around New Delhi could help determine the seasonal variation in animal bite patients, if any, in this geographical region of India. 

There are a few limitations in our study. First, the data that we analyzed were over a short span of two years and only in one hospital setting. Second, we did not have the resources to follow-up all the patients to gather information regarding their compliance to each of the vaccination regimens. Third, we did not calculate the exact wastage rates for each of the items that we considered in the final analysis. Fourth, we only considered the cost of vaccination in our analysis and not the overall cost of providing PEP. Hence, the costs of other essential items contributing to the cost of PEP such as procurement of anti-rabies serum, antiseptics, and dressing material were not considered, as done in other studies [30]. Also, we did not consider indirect costs of vaccination such as travel cost to clinic, daily wages lost, and so on, which are borne by the patients. Despite all these lacunae, the data that we presented are novel and are of great value to policy makers for making informed decisions. Future long-term studies, considering all the economic factors vital for running a successful anti-rabies program, in other high-throughput anti-rabies clinics could corroborate our findings. 

## 5. Conclusions

Following the switchover to the UTRCID regimen from the EIM regimen, a high-throughput anti-rabies clinic such as ours reduced the overall procurement cost of items required for rabies vaccination by almost 60%. Vaccine vials contributed maximally to the procurement cost. Although the consumption rate of syringes with fixed needles was slightly higher in the UTRCID regimen, as compared with the EIM regimen, it had a minor contribution to the overall procurement cost. A concerted effort to switch over to the UTRCID regimen in all high-throughput hospitals of India, catering animal bite cases could massively reduce the financial burden of running an anti-rabies program. 

## Figures and Tables

**Table 1:** Consumption pattern and procurement costs of various items required for rabies vaccination via the Essen intramuscular (EIM) regimen and updated Thai Red Cross intradermal (UTRCID) regimen.

Items	Items Consumed (EIM Regimen)	Cost per Unit (as Per 2010 Rates)	Total Procurement Cost for the EIM Regimen (as Per 2010 Rates)	Items Consumed (UTRCID Regimen)	Cost per Unit (as Per 2012 Rates)	Total Procurement Cost for the UTRCID Regimen (as per 2012 Rates)	Cost per Unit (as per 2018 Rates)	∗ Adjusted Procurement Cost for the EIM Regimen (as Per 2018 Rates)	∗ Adjusted Procurement Cost for the UTRCID Regimen (as Per 2018 Rates)
Vaccine vials	36,250	4.23	153,337.5	20,012	3.15	63,037.80	1.91	69,237.50	38,222.92
Syringes with fixed needles	36,250	0.04	1450	60,000	0.04	2400	0.04	1450	2400
Total cost	-	-	154,787.5	-	-	65,437.8	-	70,687.5	40,622.92
Cost per patient	-	-	12.27	-	-	3.87	-	5.60	2.40

Costs of items were converted from INR to USD as per historical conversion rates of 2010, 2012, and 2018, respectively; costs are expressed in USD; * values considered for final analysis and discussion.
